# Neuropeptide Y (NPY) intranasal delivery alleviates Machado–Joseph disease

**DOI:** 10.1038/s41598-021-82339-5

**Published:** 2021-02-08

**Authors:** Joana Duarte-Neves, Cláudia Cavadas, Luís Pereira de Almeida

**Affiliations:** 1 CNC - Center for Neuroscience and Cell Biology, University of Coimbra, Rua Larga, Pólo 1, Universidade de Coimbra, 3004-504 Coimbra, Portugal; 2 CIBB - Center for Innovative Biomedicine and Biotechnology, University of Coimbra, 3004-504 Coimbra, Portugal; 3 Faculty of Pharmacy, University of Coimbra, 3000-548 Coimbra, Portugal

**Keywords:** Recombinant peptide therapy, Spinocerebellar ataxia, Movement disorders, Spinocerebellar ataxia, Molecular medicine, Drug discovery, Drug delivery, Neurological disorders

## Abstract

Machado–Joseph disease (MJD) is the most common dominantly-inherited ataxia worldwide with no effective treatment to prevent, stop or alleviate its progression. Neuropeptide Y (NPY) is a neuroprotective agent widely expressed in the mammalian brain. Our previous work showed that NPY overexpression mediated by stereotaxically-injected viral vectors mitigates motor deficits and neuropathology in MJD mouse models. To pursue a less invasive translational approach, we investigated whether intranasal administration of NPY would alleviate cerebellar neuropathology and motor and balance impairments in a severe MJD transgenic mouse model. For that, a NPY solution was administered into mice nostrils 5 days a week. Upon 8 weeks of treatment, we observed a mitigation of motor and balance impairments through the analysis of mice behavioral tests (rotarod, beam walking, pole and swimming tests). This was in line with a reduction of cerebellar pathology, evidenced by a preservation of cerebellar granular layer and of Purkinje cells and reduction of mutant ataxin-3 aggregate numbers. Furthermore, intranasal administration of NPY did not alter body weight gain, food intake, amount of body fat nor cholesterol or triglycerides levels. Our findings support the translational potential of intranasal infusion of NPY as a pharmacological intervention in MJD.

## Introduction

Machado–Joseph disease or spinocerebellar ataxia type 3 (MJD/SCA3) is the most common dominantly-inherited ataxia worldwide^1^. MJD was first described in Portuguese descendants and it is highly prevalent in Azorean island of Flores, where the disease affects 1 out of 239 individuals^2^. MJD is caused by an over-repetition of the CAG trinucleotide in *ATXN3* gene leading to the synthesis of an expanded polyglutamine stretch in ataxin-3 protein^3^. Mutant ataxin-3 causes dysfunction and neurodegeneration in specific brain regions, such as cerebellum, striatum and pons^4,5^. MJD is a fatal neurodegenerative disorder, since there are no therapies available to prevent, stop or delay the disease progression.

Neuropeptide Y (NPY) is abundantly and unequally distributed throughout the mammalian brain. It is involved in several physiological functions through the activation of NPY receptors: Y1, Y2, Y4 and Y5 receptors^6^. Evidence point to several properties of NPY that have neuroprotective potential: NPY inhibits neural cell death^7^, increases neuronal trophic support^8,9^, counteracts neuroinflammation^9,10^, inhibits excitotoxicity^7,11^ and stimulates autophagy^12^. Moreover, NPY is effective in controlling and alleviating neurodegeneration in in vitro and in vivo models of different brain diseases, including Alzheimer’s disease, Parkinson’s disease and Huntington’s disease^8,13,14^.

Our recent findings demonstrated for the first time that NPY levels are reduced in MJD patients and mouse models and that NPY has therapeutic potential in MJD mice^9^. Indeed, NPY overexpression mediated by adeno-associated viral (AAV) vectors was able to reduce motor impairments and neuropathology of MJD mouse models. Nevertheless, stereotaxic injection of viral vectors is an invasive strategy and presents many safety and regulatory restrains to its use in the clinics. The intranasal route, which constitutes a more direct route towards the brain, was successfully used to deliver NPY to rodents^15,16^. Moreover, the safety of the intranasal administration of NPY to humans^17^ and the therapeutic potential in posttraumatic stress disorder^18,19^ have already been evaluated in clinical trials.

Therefore, we aimed to explore this promising NPY therapy by using a non-invasive and more translational approach: delivery of NPY to MJD mice through the intranasal route. For the first time, we show mitigation of motor and balance impairments and neuropathology in MJD mice resulting from intranasal administration of NPY.

## Results

### Intranasal administration of NPY alleviates balance and motor coordination impairments of MJD transgenic mice

We previously showed that MJD transgenic mice exhibits a severe ataxic phenotype^20^ and that cerebellar NPY overexpression was able to improve this motor coordination and balance impairments^9^. Considering stereotaxic injections are an invasive approach, we investigated whether intranasal NPY administration, a strategy more amenable to translation to increase cerebral NPY levels, would replicate this neuroprotective effect. Thus, we administered intranasally a NPY solution, or vehicle in the control animals, to 5-week-old MJD transgenic mice, 5 days a week for a total of 8 weeks. At this time-point animals already exhibit a relatively mild motor behavior phenotype that further evolves over the following 8 weeks. At 4 and 8 weeks after the beginning of treatment, we performed behavioral tests to evaluate balance and motor coordination.

**Figure 1:**
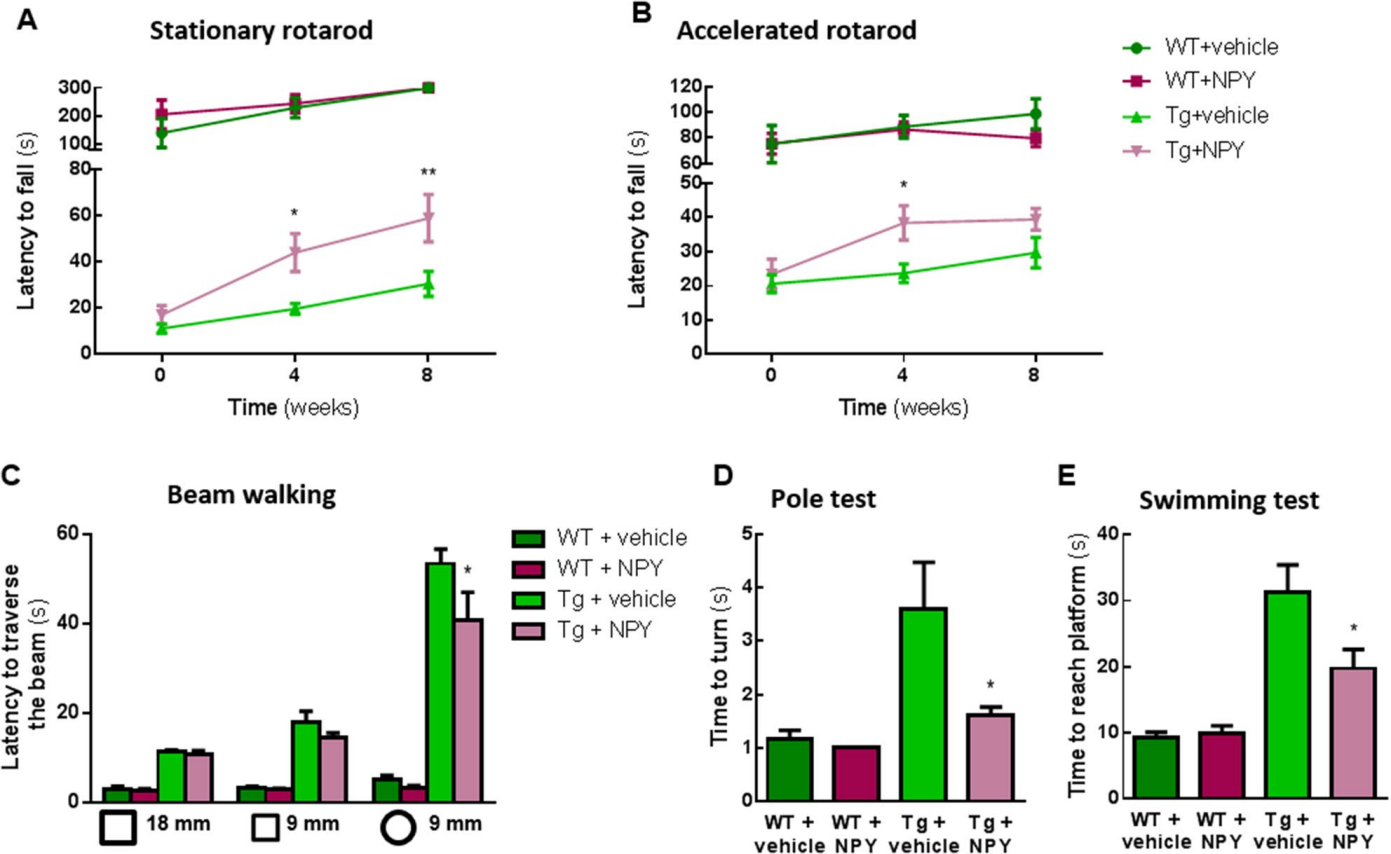
Intranasal administration of NPY alleviates balance and motor coordination impairments of MJD transgenic mice. (**A,B**) NPY treated MJD transgenic mice presented a better performance either in the stationary (5 r.p.m) and in the accelerated (from 4 to 40 r.p.m. in 5 min) rotarod test. (**C**) Beam walking test showed that 8 weeks of NPY intranasal administration was able to improve the ability of transgenic mice to traverse the round beam. (**D**) In the vertical pole test, NPY-administered transgenic mice turned downwards easier than transgenic mice administered with vehicle, 8 weeks after treatment beginning. (**E**) At 8 weeks of treatment, NPY-treated Tg mice had a better performance in the swimming test, since they took less time to reach the platform at the end of swimming pool. Data are expressed as mean ± SEM. Statistical significance was evaluated with two-way ANOVA (**A**–**C**) followed by Sidak’s post hoc test and with one-way ANOVA (**D**,**E**) followed by Tukey’s post hoc test; *p < 0.05 and **p < 0.01 compared with Tg + vehicle; n = 10.

Motor coordination was assessed using constant velocity rotarod test which showed that 4 and 8 weeks of NPY intranasal administration increased the Tg mice’s latency to fall off the rod (Fig. 1A, 19.60, *p < 0.05 and **p < 0.01 relative to Tg + vehicle, n = 10). Moreover, this beneficial effect of NPY administration was also observed in the accelerated rotarod test, since Tg mice treated with NPY presented an improved performance at 4 weeks compared to control animals (Fig. 1B, *p < 0.05, n = 10).

Balance and motor coordination were also measured by the latency time for each animal to traverse a series of progressively more difficult beams of square and round cross-section, in the beam walking test. Eight weeks after beginning of NPY administrations, Tg mice took less time to cross the round beam when compared with mice administered with a vehicle solution (Fig. 1C, *p < 0.05, n = 10).

This phenotypic improvement was further investigated in the vertical pole test. MJD transgenic mice in the control group displayed increased time to orient downward in the vertical pole, when compared to transgenic mice treated with NPY for 8 weeks (Fig. 1D, *p < 0.05, n = 10).

Furthermore, at 8 weeks of treatment with NPY, transgenic mice swam faster and presented less difficulties to reach the platform in the swimming test, when compared to control transgenic animals (Fig. 1E, *p < 0.05, n = 10).

Taken together, these data indicate that intranasal administration of NPY efficiently rescues motor coordination and balance impairments of MJD transgenic mice.

### Intranasal administration of NPY reduces cerebellar neuropathology

To assess whether phenotypic improvements correlated with histopathological alterations in the cerebellum, we compared histological parameters and further evaluated the effects of intranasal NPY treatment. For that purpose, after 8 weeks of NPY or vehicle treatment, MJD Tg mice were sacrificed and cerebellar layers thickness, number of Purkinje cells and number of mutant ataxin-3 aggregates in these cells were quantified in cerebella of mice from the different treatment groups.

**Figure 2:**
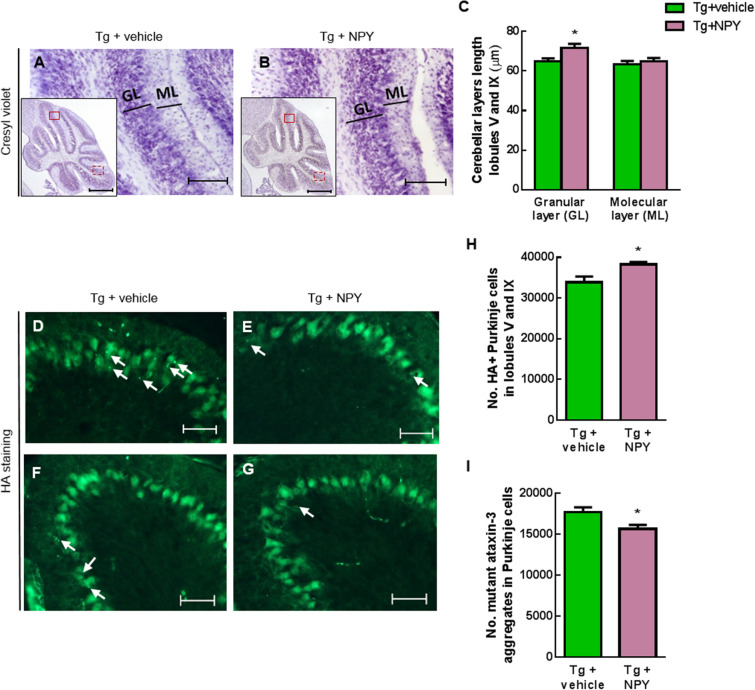
Intranasal administration of NPY reduces cerebellar neuropathology. Eight weeks after beginning of intranasal NPY administration MJD Tg animals were sacrificed for histological analysis. (**A**,**B**) Sagittal cresyl violet-stained sections displaying cerebellar granular and molecular layers from Tg + vehicle (**A**) and Tg + NPY (**B**). Quantification analysis of the granular and molecular layers length revealed NPY treatment prevented the reduction of granular layer thickness in lobules V and IX ((**C**), *p < 0.05, n = 10), while no difference in molecular layer thickness was observed. (**D**–**G**) Immunostaining of mutant ataxin-3 with an anti-HA antibody, revealing Purkinje cells. Tg mice treated with intranasal NPY presented a higher amount of Purkinje cells (*p < 0.05, n = 5–8) than controls, as quantified in (**H**). Some Purkinje cells exhibited mutant ataxin-3 aggregates (arrows). A reduction in the number of aggregates in Purkinje cells was observed in Tg treated with NPY when compared to control animals (*p < 0.05, n = 6–9), as quantified in (**I**). Data are expressed as mean ± SEM. Statistical significance was evaluated with two-way ANOVA followed by Sidak’s post hoc test (**C**) and with unpaired Student’s t-test (**H**,**I**). Scale bars: 500 μm and magnification 100 μm (**A**,**B**), and 50 μm (**D**–**G**).

To examine morphological abnormalities, cresyl violet-stained sagittal sections were used (Fig. 2A,B). Tg mice treated with intranasal NPY showed a larger granular layer thickness, measured in lobules V and IX, compared with Tg + vehicle mice (Fig. 2C, *p < 0.05, n = 10). No difference was found in molecular layer thickness between animals in both treatment groups.

Since in this transgenic mouse model, HA-tagged mutant ataxin-3 was expressed in cerebella, mainly in Purkinje cells where it aggregates^21^, we immunolabeled Purkinje cells with an anti-HA antibody (Fig. 2D–G). Upon quantification we found that the number of Purkinje cells in lobules V and IX was higher in the cerebella of NPY treated Tg mice, compared with control animals (Fig. 2H, *p < 0.05, n = 5–8).

One of the main hallmarks of MJD is aggregation of mutant ataxin-3 in cerebellar neurons^22,23^. Hence, we investigated whether the beneficial effects of intranasal NPY correlated with reduction of mutant ataxin-3 aggregate numbers in Tg mice cerebella. Eight weeks of NPY treatment mediated decrease of the number of aggregates present in Purkinje cells (Fig. 2I, *p < 0.05, n = 6–9).

Altogether, these results suggest NPY intranasal administration preserves cerebellar structure and decreases the number of mutant ataxin-3 aggregates in Purkinje cells.

### Intranasal administration of NPY does not affect body weight, food intake levels, white adipose tissue and dyslipidemia parameters of MJD Tg and WT mice

NPY is an orexigenic peptide playing an important role in controlling food intake and body weight, especially due to its functions in hypothalamic mechanisms of energy homeostasis^24–26^.

**Figure 3:**
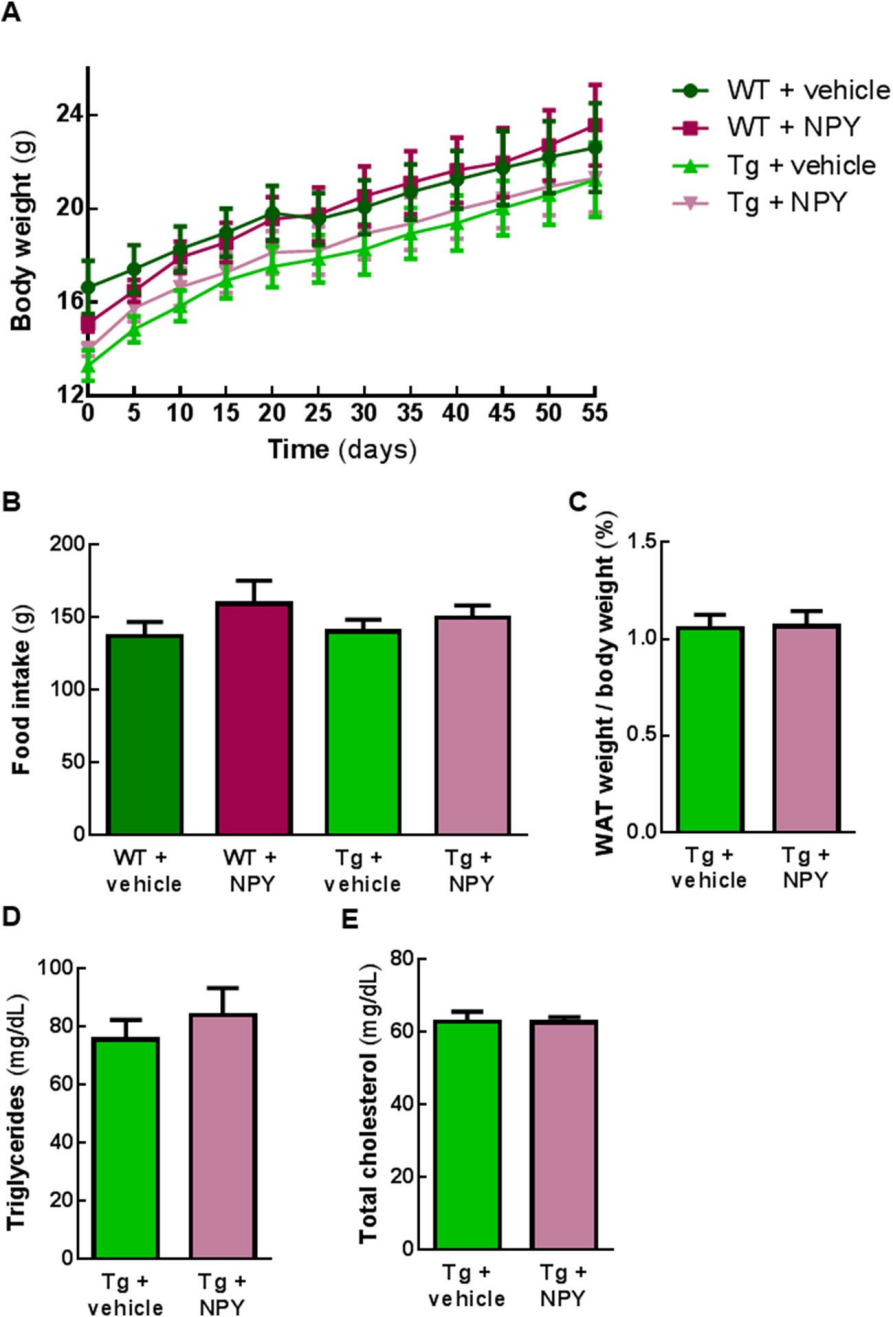
Intranasal administration of NPY does not affect body weight, food intake levels, white adipose tissue weight and lipidic parameters of MJD Tg and WT mice. (**A**) For 8 weeks, every 5 days, MJD Tg and WT littermates mice were weighted. No significant differences were observed between NPY-treated and vehicle-administrated animals (p > 0.05, n = 6–10). (**B**) Food intake was measured for 7 weeks after the beginning of treatment. No significant changes in caloric intake were observed between animal groups (p > 0.05, n = 4–8). (**C**) The ratio between white adipose tissue weight and body weight does not differ in mice treated with NPY compared with control mice (p > 0.05, n = 10). (**D**,**E**) Serum analysis of Tg + vehicle and Tg + NPY, 8 weeks after first intranasal administration, revealed no changes in triglycerides (**D**) nor in total cholesterol (**E**) levels between experimental groups (p > 0.05, n = 10). Data are expressed as mean ± SEM. Statistical significance was evaluated with two-way ANOVA followed by Tukey’s post hoc test (**A**), one-way ANOVA followed by Bonferroni’s post-hoc test (**B**) and with unpaired Student’s t-test (**C**–**E**).

To investigate the impact of NPY intranasal treatment on body weight, we measured MJD Tg and WT mice weight for 8 weeks. NPY treated Tg and WT mice did not present significant body weight differences compared to control groups (Fig. 3A, p > 0.05, n = 6–10). This correlated with a lack of intranasal NPY ability to stimulate food intake. In fact, there were no significant differences in food intake between treated and non-treated, transgenic or wild-type animal groups (Fig. 3B, p > 0.05, n = 4–8). Moreover, the amount of white adipose tissue was not increased in Tg mice treated with intranasal NPY, compared to Tg + vehicle (Fig. 3C, p > 0.05, n = 10).

Furthermore, serum analysis was performed to evaluate possible alterations consistent with obese phenotype usually observed in mice overexpressing NPY^25^. The levels of cholesterol and triglycerides were not statistically different from control group (Fig. 3D,E, p > 0.05, n = 10).

These evidence suggest intranasal administration of NPY does not impact on energy homeostasis in this MJD Tg mice nor in WT littermates.

## Discussion

To the best of our knowledge, we show, for the first time, mitigation of motor and balance impairments and neuropathology in MJD mice upon intranasal administration of NPY, without observable side effects.

Even though Machado–Joseph disease is characterized by neurodegeneration in different brain regions, the cerebellum is the brain region most affected and cerebellar damages are associated with ataxia^5^. Therefore, in the present study, we used a transgenic mouse model that presents cerebellar neuropathological and motor features resulting from expression of an ataxin-3 C-terminal fragment carrying the mutant polyglutamine expansion under control of the L7-promoter^21^. Despite probably being the most severe mouse model of Machado–Joseph disease available, intranasally-administered NPY was able to significantly mitigate its motor impairments. We found that intranasal administration of NPY robustly improved the performance of MJD transgenic mice in the stationary and accelerated rotarod, beam walking test, pole test and swimming test. Moreover, we found Purkinje cell preservation in NPY-treated transgenic mice in parallel with decrease in mutant ataxin-3 aggregate number. Reduction of mutant ataxin-3 aggregation and associated toxicity, may explain the preserved larger granular layer thickness of NPY-treated mice in comparison with control animals.

These results are in line with our previous published data, in a study in which we overexpressed NPY in cerebellum and striatum of MJD mouse models^9^. Our results suggested that NPY neuroprotective effects were due to an increase of brain-derived neurotrophic factor (BDNF), an important survival agent, and a decrease of neuroinflammation. More recently, Fatoba et al.^27^ also demonstrated that intranasal administration of NPY to Huntington’s disease mice was able to attenuate inflammation and increase BDNF expression, through the activation of Y_2_ receptors. Thus, we hypothesize that NPY intranasal administration rescues neurons from degeneration by enhancing trophic support and attenuating inflammatory pathways in MJD mice. On the other hand, we should also consider other described protective mechanisms in which NPY plays a role, such as stimulation of autophagy to promote the clearance of disease-causing protein aggregates, suppression of excitotoxicity and regulation of calcium homeostasis (reviewed in^28^). Nonetheless, further studies should be pursued to clarify the neuroprotective mechanisms underlying NPY effects on MJD, as well as the role of each NPY receptor.

NPY is a potent orexigenic agent (stimulates feeding), playing an important role in physiologic control of food intake and body weight^29^. Indeed, sustained up-regulation of hypothalamic NPY in rodent models is associated to a disruption of feeding patterns, eliciting a strong feeding response^25^. Nonetheless, in our study, intranasal administration of NPY did not affect body weight, food intake levels, white adipose tissue weight nor dyslipidemia parameters, such as cholesterol and triglycerides serum levels, of mice. These results are in line with other studies in which intranasal infusion of NPY did not influence the body weight gain in rodents^16,27,30^. Possibly, intranasal NPY is not reaching hypothalamus in sufficient concentration to interfere with hypothalamic mechanisms of energy regulation.

The transgenic mice used in these experiments express only a truncated C-terminal fragment of mutant ataxin-3, opening the possibility for this mouse model to represent a wider model of polyQ-associated spinocerebellar ataxias. Therefore, considering that polyQ diseases share many pathogenic mechanisms, NPY treatment mediated by intranasal administration may be a therapeutic strategy for other polyQ-associated ataxias. Moreover, it was already described a widespread distribution of NPY to different rodent brain regions after intranasal infusion, including the olfactory bulb, striatum, hippocampus, amygdala, hypothalamus and cerebellum^27,31^, allowing NPY neuroprotective effects to take place in brain regions affected by each disease. Indeed, it was previously shown that intranasal NPY ameliorates motor and neuropathology deficits and extends life span of Huntington’s disease transgenic mice^27^.

Intranasal administration provides a non-invasive strategy to deliver drugs to CNS bypassing the blood brain barrier. After administration, peptides such as NPY rapidly reach several brain regions, through pathways along olfactory and trigeminal nerves innervating the nasal cavity^32^. Moreover, intranasal infusion can be performed without the need of anaesthetics in conscious animals or humans, making this route of administration more clinically suitable. Previous studies have already administered NPY through intranasal infusion to humans^33,34^. Furthermore, this therapeutic strategy has been tested in clinical trials in patients with post-traumatic stress disorder and acute stress disorder^17–19^.

Our findings support the translational potential of intranasal infusion of NPY as a pharmacological intervention in MJD patients, for whom there is no effective treatment to reverse, stop or prevent disease progression.

## Methods

### Animals

C57Bl/6-background transgenic mouse model expressing the C-terminal-truncated ataxin-3 with 69 glutamine repeats and an N-terminal haemagglutinin (HA) epitope driven by Purkinje-cell-specific L7 promoter were obtained from parallel breeding at our research center (CNC) of a colony of transgenic mice initially obtained from Gunma University Graduate School of Medicine^21^. Genotyping was performed by PCR. Gender- and age-matched transgenic (Tg) and wild type (WT) littermates at 5 weeks of age were used in this study.

Body weight of each mouse was measured 5 days a week, during 7 weeks. Food intake was measured twice a week, by weighing the amount of food ingestion in each cage (each cage housed 2 or 3 mice within the same treatment group).

All experimental protocols were approved by ORBEA (Órgão Responsável pelo Bem-Estar dos Animais da Faculdade de Medicina da Universidade de Coimbra e do Centro de Neurociências e Biologia Celular) and performed in accordance with the European Community directive (2010/63/EU) covering the protection of animals used for scientific purposes. All researchers of the present study received proper training (FELASA-certified course) and certification from the Portuguese authorities (Direcção Geral de Alimentação e Veterinária).

### Intranasal administration of NPY

Lyophilized neuropeptide Y tagged with rhodamine B (Rhodamineas-Tyr-Pro-Ser-Lys-Pro-Asp-Asn-Pro-Gly-Glu-Asp-Ala-Pro-Ala-Glu-Asp-Leu-Ala-Arg-Tyr-Tyr-Ser-Ala-Leu-Arg-His-Tyr-Ile-Asn-Leu-Ile-Thr-Arg-Gln-Arg-Tyr-NH2; Synpeptide Co., China) was ressuspended in 2% acetonitrile/distilled water.

Using the intranasal grip as previously described^35^, each mouse was restrained and held with their head neck parallel to the floor while 10 µl of NPY solution (0.25 mg/kg of body weight/day), or vehicle in the control group, was infused into the nares with a pipetman and disposable plastic tip, 5 days a week, during 8 weeks. Care was taken to avoid direct tip contact with intranasal mucosa. After administration the head of the mouse was held in the same position for approximately 10 s to prevent loss of the solution from the nares.

### Behavioral assessment

Mice were subjected to locomotor tests before and 4 and 8 weeks after the beginning of treatment with NPY. As in our previous work, animals were acclimatized for 1 h to a quiet room with controlled temperature and ventilation, dimmed lighting, and handled prior to behavioral testing^9^.

#### Stationary and accelerated rotarod

Motor coordination and balance were assessed using rotarod apparatus (Letica Scientific Instruments, Panlab, Spain). In stationary rotarod test, the drum was rotating at a constant speed of 5 rpm, over a period of 5 min. In the accelerated rotarod test, mice were placed in a rod at an accelerated speed, from 4 to 40 rpm over a period of 5 min. The time during which mice remain walking in the rotation drum was recorded. For each test, sessions consisting of three trials per day with a 20-min inter-trial interval were carried out and the mean of the trials was averaged.

#### Beam walking

As previously described^9^, motor coordination and balance of mice were assessed by measuring the ability of the mice to traverse a graded series of narrow beams to reach an enclosed safety platform. The beams consisted of long strips of wood (1 m) with an 18- or 9-mm square wide and a 9-mm round diameter cross-sections, placed horizontally, 25 cm above the bench surface. Mice performed two consecutive trials on each beam, progressing from the widest to the narrowest and the square to the round beams, and the mean latency time to traverse the beam was taken to analysis. Any animal that did not cross within 60 s was assigned a maximum value of 60 s for analysis.

#### Pole test

The vertical pole test was used to assess motor coordination and balance of mice. As previously described^36^, each mouse was positioned head-upward on the top of a round-surfaced pole (with 52 cm of height and 1 cm of diameter). The time to orient downward (time to turn) was recorded and the maximum observation time defined was 2 min. Five consecutive trials were performed with an inter-trial interval of 60 s and the best time was considered.

#### Swimming test

A rectangular aquarium (length: 70 cm; height: 20 cm; width: 15 cm) with a platform located at 9 cm upper the floor was filled up to the area of the platform with water at approximately 23 °C. Each mouse was placed in the aquarium, in the opposite side of the platform and the time that mice took to reach the platform was recorded. The time of three trials with an inter-trial interval of 60 s was recorded and the results express the average value of the three trials.

### Collection of blood and tissues

After being anesthetized with a mixture of ketamine (100 mg/kg) with xylazine (10 mg/kg) through an intraperitoneal injection, animals were intracardially perfused with PBS and 4% paraformaldehyde (PFA; Sigma-Aldrich, USA). Brains were removed, post-fixed in 4% PFA for 24 h, cryoprotected by incubation in 25% sucrose/PBS for 48 h and stored at − 80 °C. Gonadal white adipose tissue was isolated and weighted.

Prior to perfusion, blood was collected and serum was isolated by centrifugation (2000×*g*, 15 min). Tryglicerides and total cholesterol were quantified in the serum.

### Immunohistochemical procedure

Sagittal sections of 35 µm thickness were cut using a cryostat (LEICA CM3050S, Germany) at − 21 °C, collected in anatomical series and stored in 48-well trays as free-floating sections in PBS supplemented with 0.05 µM sodium azide (Sigma-Aldrich), at 4 °C.

As previously described^9^, double staining for HA (mouse monoclonal anti-haemagglutinin; 1:1000; InvivoGen, France) and nuclear marker [4′,6-diamidino-2-phenylindole (DAPI); Sigma-Aldrich] was performed. After RT 1 h incubation with blocking solution, and overnight 4 °C incubation with primary antibody, sections were washed and incubated for 2 h at RT with the corresponding secondary antibody coupled to fluorophores (1:200; Molecular Probes, Life Technologies, USA) diluted in the respective blocking solution. The sections were then mounted in Mowiol reagent (Sigma-Aldrich) on microscope slides.

Staining was visualized using Zeiss Axioskop 2 plus imaging microscope (Carl Zeiss Microimaging, Germany) using 5×, 20× and 40× objectives and the AxioVision 4.7 software package (Carl Zeiss Microimaging, https://www.zeiss.com/microscopy/int/products/microscope-software.html). Quantitative analysis was performed with a semiautomated image-analysis software package (ImageJ 1.48q software, USA, https://imagej.net/Welcome).

### Cresyl violet staining

As previously described^9^, premounted sagittal sections were stained with cresyl violet (Sigma-Aldrich) for 2 min, differentiated in 70% ethanol, dehydrated by passing through 95% ethanol, 100% ethanol and xylene solutions, and mounted onto microscope slides with Eukitt.

### Quantification of granular and molecular layers thickness

Lobules V and IX granular and molecular layers thickness was assessed by the mean of three different measures to each layer, in three sections at 280 µm intervals, including the most central one (lateral 0 mm)^37^, scanned with a 20× objective. Layers thickness was assessed near the apex of V and IX lobules. Measurements were performed using image-analysis software (ImageJ 1.48q software, USA, https://imagej.net/Welcome).

### Cell counts of Purkinje cells in cerebellar lobules V and IX and mutant ataxin-3 inclusions in Purkinje cells

Three sequential sagittal sections at 280 μm intervals encompassing the hemicerebellar lobules V and IX were scanned with a 20× objective. All Purkinje cells in the lobules V and IX were manually counted, and its number extrapolated for the entire cerebellum.

Eight sagittal sections spread over the lateral extent of cerebellum were scanned with a 20× objective. All HA aggregates in Purkinje cells were manually counted and the average number of inclusions was extrapolated to the whole cerebellum.

The total number of cells/aggregates was determined with the following formula: (S1 + S2 + S3 + …) × 3 or 8 (cells or aggregates) × 2, in which S1 is the number in section 1, S2 number in section 2, etc., encompassing sections at 280 µm intervals and both cerebellar hemispheres.

### Statistical analysis

Data are expressed as mean ± standard error of the mean (SEM). Statistical comparisons were performed using either an unpaired Student’s t test or 1-way or 2-way ANOVA followed by Sidak, Tukey or Bonferroni post-hoc tests, to address the effects of treatment (intranasal administration of NPY) on mice groups. Significance thresholds were set at p < 0.05, p < 0.01 or p < 0.001.

## Abbreviations

MJD: Machado–Joseph disease
NPY: Neuropeptide Y
Tg: Transgenic
WT: Wild-type
